# FGF23 Induction of O-Linked N-Acetylglucosamine Regulates IL-6 Secretion in Human Bronchial Epithelial Cells

**DOI:** 10.3389/fendo.2018.00708

**Published:** 2018-11-27

**Authors:** Stefanie Krick, Eric Scott Helton, Samuel B. Hutcheson, Scott Blumhof, Jaleesa M. Garth, Rebecca S. Denson, Rennan S. Zaharias, Hannah Wickham, Jarrod W. Barnes

**Affiliations:** ^1^Division of Pulmonary, Allergy and Critical Care Medicine, School of Medicine, University of Alabama at Birmingham, Birmingham, AL, United States; ^2^Hillel Connections Program, Bloom Hillel, University of Alabama, Tuscaloosa, AL, United States

**Keywords:** O-GlcNAc, FGF23 = fibroblast growth factor 23, NFAT (nuclear factor expression of activated T cell), IL-6 (Interleukin 6), inflammation

## Abstract

The hexosamine biosynthetic pathway (HBP) generates the substrate for the O-linked β-N-acetylglucosamine (O-GlcNAc) modification of proteins. The HBP also serves as a stress sensor and has been reported to be involved with nuclear factor of activated T-cells (NFAT) activation, which can contribute to multiple cellular processes including cell metabolism, proliferation, and inflammation. In our previously published report, Fibroblast Growth Factor (FGF) 23, an important endocrine pro-inflammatory mediator, was shown to activate the FGFR4/phospholipase Cγ (PLCγ)/nuclear factor of activated T-cells (NFAT) signaling in chronic inflammatory airway diseases such as cystic fibrosis (CF) and chronic obstructive pulmonary disease (COPD). Here, we demonstrate that FGF23 increased the O-GlcNAc modification of proteins in HBECs. Furthermore, the increase in O-GlcNAc levels by FGF23 stimulation resulted in the downstream activation of NFAT and secretion of interleukin-6 (IL-6). Conversely, inhibition of FGF23 signaling and/or O-GlcNAc transferase (OGT)/O-GlcNAc reversed these effects. Collectively, these data suggest that FGF23 induced IL-6 upregulation and secretion is, at least, partially mediated via the activation of the HBP and O-GlcNAc levels in HBECs. These findings identify a novel link whereby FGF23 and the augmentation of O-GlcNAc levels regulate airway inflammation through NFAT activation and IL-6 upregulation in HBECs. The crosstalk between these signaling pathways may contribute to the pathogenesis of chronic inflammatory airway diseases such as COPD and CF as well as metabolic syndromes, including diabetes.

## Introduction

Human fibroblast growth factors (FGFs) are classified as intracrine, paracrine, and endocrine FGFs depending on their action process with endocrine FGFs playing key roles in metabolism including bile acid, energy, and phosphate/active vitamin D metabolism (1, 2). FGF23 is a 27-kDa protein that has been shown to be strongly associated with the risk of chronic kidney disease progression, systemic inflammation, and mortality (3, 4). Our recent data characterized FGF23 signaling as an important mediator in inflammatory airway diseases such as cystic fibrosis (CF) and chronic obstructive pulmonary disease (COPD) (5, 6). In the COPD lung, FGF23 activated the phospholipase Cγ (PLCγ)/nuclear factor of activated T-cells (NFAT) signaling pathway leading to airway inflammation(5).

NFAT signaling has also been linked to inflammatory cytokine production in hepatocytes, angiogenesis, cardiomyocyte hypertrophy, and many other biological processes (7–10). On a molecular level, NFAT is regulated by the phosphatase calcineurin, which dephosphorylates NFAT and triggers cytoplasmic to nuclear translocation. Upon nuclear translocation, NFAT interacts with multiple factors to regulate gene expression of molecules involved in the aforementioned disease processes.

Although the activation of the PLCγ/NFAT signaling pathway by FGF23 has been studied, the downstream molecules that are affected have not been fully characterized. Several reports have shown that the activation of the hexosamine biosynthetic pathway (HBP) (11, 12), a stress sensor, is linked to NFAT activation and may have a definitive role in inflammation (13, 14). It is well-documented that the HBP serves as a precursor for several glycosylation pathways (14–16). Once activated, the HBP generates the sugar nucleotide UDP-N-acetyl-glucosamine (UDP-GlcNAc), which is a substrate for hyaluronan (HA), N-linked glycosylation, and for the O-linked β-N-acetylglucosamine (O-GlcNAc) modification of proteins (17). The O-GlcNAc modification is a single monosaccharide addition to proteins at unoccupied serine and threonine residues and is similar to protein phosphorylation. Addition or removal of O-GlcNAc is a dynamic process that is regulated by the glycosyltransferase OGT (O-GlcNAc transferase) and the glycosyl hydrolase OGA (β-N-acetylglucosaminidase), each of which serves as a stress sensor and flux mediator (16, 18, 19) in response to changes in the cellular microenvironment (i.e., stress stimuli) (20, 21). An imbalance in the OGT/O-GlcNAc axis has been shown to regulate several cellular functions including cell cycle and proliferation, cardiac hypertrophy, cell metabolism, and inflammation (22, 23). We have previously shown that OGT expression/activity was elevated in the pulmonary vascular disease, pulmonary arterial hypertension, regulated pulmonary arterial smooth muscle cell proliferation, and was associated with clinical disease worsening (24). However, its role in chronic inflammatory airway diseases has not been determined. Furthermore, the role the O-GlcNAc modification on PLCγ/NFAT signaling in airway inflammation, specifically upon FGF23 activation, has not been established. Here, we demonstrate that FGF23 stimulates the O-GlcNAc stress response in human bronchial epithelial cells, which is essential for NFAT transcriptional regulation of the inflammatory cytokine, IL-6, and may be involved in chronic inflammatory airway diseases such as COPD and CF.

## Materials and methods

### Cell culture, reagents, and treatment conditions

16HBE cells (or HBECs), a SV40-immortalized human bronchial epithelial cell line, were grown on plates, coated with Collagen IV (6.5 μg/cm2; Sigma; St. Louis, MO), in medium consisting of Eagle's Minimum Essential Medium (EMEM) supplemented with 10% heat-inactivated fetal bovine serum (Atlas Biologicals; Fort Collins, CO) and without antibiotics as shown previously (5). Human recombinant FGF23 was utilized at 20 ng/ml and stocks were prepared in sterile PBS containing 0.1% BSA as recommended by the manufacturer (PeproTech; Rocky Hill, NJ). Where indicated, a 1 h pretreatment for all inhibitors, including the PLCγ inhibitor (U73122; 0.1 mM and 1.0 mM); FGFR4 inhibitor (100 nM R4 final; BLU9931; Selleck Chemicals; Houston, TX); Cyclosporine, a calcineurin inhibitor that down-regulates NFAT activation (CsA; 100 nM final); Thiamet G, an OGA inhibitor (TG; 25 nM final); and OSMI-1, an OGT inhibitor (25 μM final), was performed prior to the addition of FGF23, all incubated for 24 h. Unless indicated otherwise, all inhibitors were purchased from Sigma (St. Louis, MO).

### Western immunoblotting and antibodies

Cell lysates were prepared in RIPA buffer with 1x protease and phosphatase inhibitor cocktails (RPI; Mount Prospect, IL), PUGNAC (50 μM; Sigma, St. Louis, MO), and Thiamet G (25 μM, Sigma) added to block removal of the O-GlcNAc modification and subjected to immunoblotting as previously described (24). Briefly, nitrocellulose membranes were probed with antisera for the following: (1) anti-mouse O-GlcNAc (1:1,000; clone CTD 110.6 Biolegend, San Diego, CA, USA), anti-rabbit OGT (1:5,000, Sigma), anti-rabbit OGA (1:5,000 Bethyl Laboratories, Montgomery, TX, USA), anti-rabbit phospho-PLCγ (8713S) (Cell Signaling, Danvers, MA, USA), anti-rabbit PLCγ (2822S) (Cell Signaling), anti-rabbit phospho-ERK (9101S) (Cell Signaling), anti-rabbit ERK (4695S) (Cell Signaling), and anti-mouse β-actin (Sigma). Probed blots were developed using enhanced chemiluminescence Supersignal Femto Substrate (Thermo Scientific; Grand Island, NY, USA). All blots were imaged using the GE Imaging System (GE Healthcare, USA) and densitometric analyses was performed using Image J (25).

### IL-8 and IL-6 ELISA and mRNA assessment

IL-8 and IL-6 enzyme-linked immunosorbent assays (ELISA) from Invitrogen (Thermo Scientific) were used according to the manufacturer's protocol. HBECs were stimulated for 24 h with FGF23, TG, or OSMI-1, and 100 μl of the medium (undiluted) was used for measurement.

RNA was extracted using the GeneJET RNA purification kit (Thermo Scientific, Grand Island, NY, USA). For gene expression analysis, qPCR was performed by using Taqman probes (Life technologies/Applied Biosystems, Carlsbad, CA, USA) with the following: Hs00174103_m1 for IL-8, Hs00174131_m1 for IL-6, and Hs02758991_g1 for GAPDH.

### Gene silencing of OGT and NFAT isoforms using siRNA

16HBE cells were used for NFAT and OGT siRNA-mediated knockdown (KD) experiments as previously described (5, 25). Briefly, 5 × 10^4^ cells were seeded in coated 24-well plates and transfected for 6 h in 0.5 mL OptiMEM with 5 nmol of either AllStar negative control or OGT siRNAs (Thermo Scientific, Grand Island, NY, USA) or NFATC2 (NFAT2c) or NFATC3 (NFAT3c) siRNAs (Qiagen; Hilden, Germany) using 1.5 μL/well of Qiagen HiPerFect transfection reagent. Following the transfection, medium was replaced with complete medium and the cells were subjected to an additional 48 h incubation to allow for NFAT or OGT knockdown. After a 24 h treatment with FGF23, wells were washed with 1.0 mL cold phosphate buffered saline (pH 7.4) and RNA was extracted using the GeneJET RNA purification kit (Thermo Scientific, Grand Island, NY, USA). For some experiments, HBECs were transfected with siRNA against OGT or NFAT2c/3c and 100 ul of conditioned medium was collected for ELISA.

### NFAT luciferase reporter assay

As described previously (5), 16HBE cells plated at 1.5 × 10^4^ per well in a coated 96-well plate were transfected with 100 ng of DNA mixture containing the constitutively-active Renilla-luciferase construct, as a transfection control, together with a Fire Fly Luciferase reporter construct, and a NFAT reporter construct serving provided with the NFAT Cignal Reporter Assay Kit (Qiagen; Hilden, Germany). Transfection was performed overnight under serum-free conditions in OptiMEM using Lipofectamine 2000 transfection reagent (Thermo Scientific, Grand Island, NY, USA). Cells were collected after an additional 24 h treatment and a Luciferase assay was performed using the Dual-Luciferase Reporter Assay System (Promega; Madison, WI, USA) as directed by the manufacturer (Promega; Madison, WI, USA). Relative light units (RLUs) were measured utilizing a SpectraMax i3x plate reader equipped with dual injectors (Molecular Devices; Sunnyvale, CA, USA).

### Statistics

Data were analyzed with Prism5 (GraphPad Software, Inc., La Jolla, CA) and shown as mean ± SEM using Student's *t*-test and analysis of variance or Kruskal–Wallis *H*-test with one-way ANOVA with appropriate post tests for at least three independent experiments. Significance was accepted at *p* < 0.05.

## Results

### FGF23 stimulates the HBP/O-GlcNAc modification of proteins in human bronchial epithelial cells

**Figure 1:**
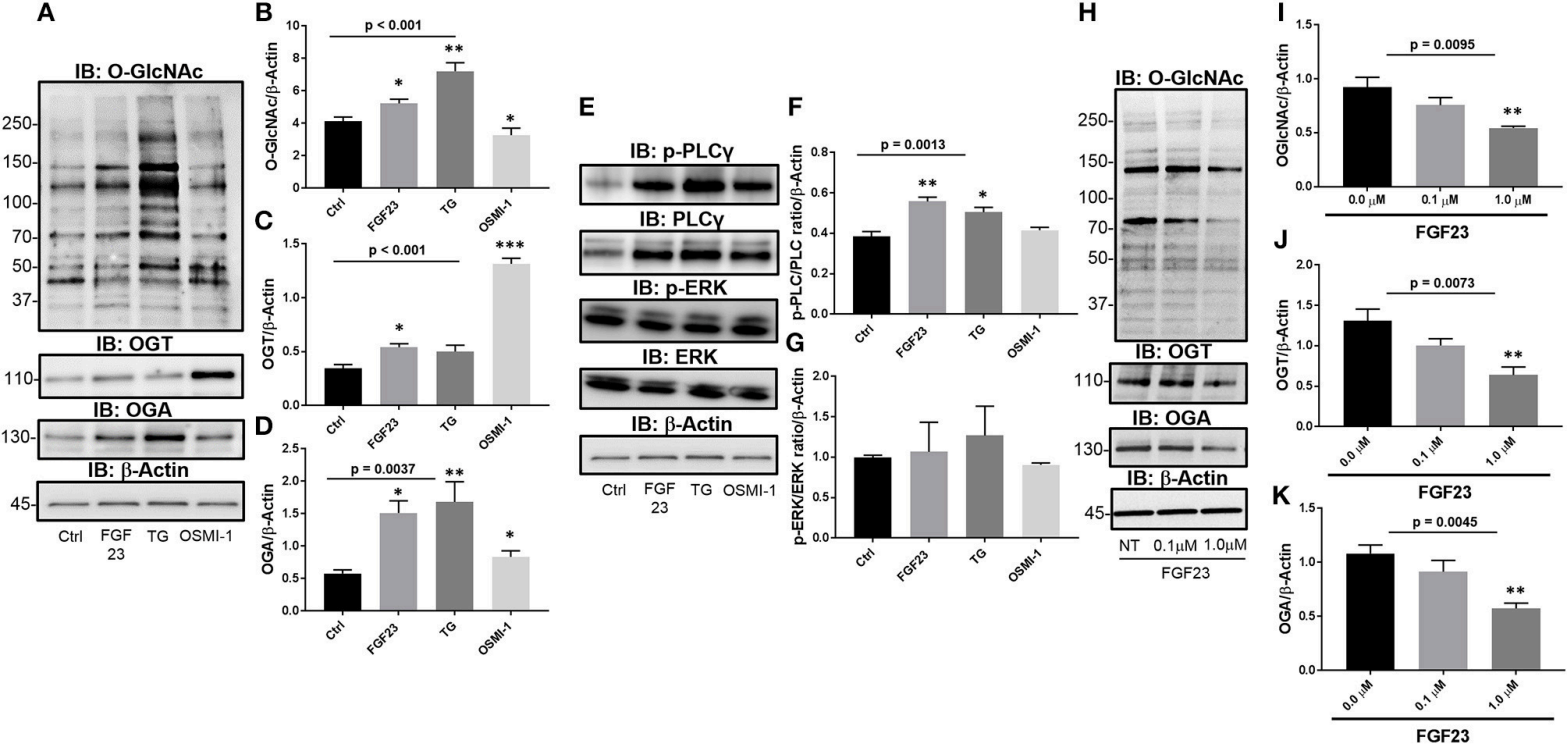
FGF23 stimulates the HBP/O-GlcNAc modification of proteins via the PLCγ signaling pathway in human bronchial epithelial cells. **(A)** Representative Immunoblots showing global O-GlcNAc, OGT, OGA, and β-Actin from HBECs treated as described. **(B–D)** Densitrometric quantitation of O-GlcNAc, OGT, and OGA from **(A)**. **(E)** Representative Immunoblots showing phosphorylation of PLCγ and ERK, total PLCγ and ERK, and β-Actin from HBECs treated as described. **(F,G)** Densitrometric quantitation of Immunoblots from **(A)**. **(H)** Representative Immunoblots of global O-GlcNAc, OGT, OGA, and β-Actin from HBECs treated with a PLCγ inhibitor (U73122 at 0, 0.1, and 1.0 μM) and FGF23 (20 ng/ml) for 24 h. **(I–K)** Densitrometric quantitation of O-GlcNAc, OGT, and OGA from **(H)**. Western blots were performed as triplicates of the same experiment. Statistical analysis was done using ANOVA or Student's *t*-test showing means ± S.E.M. with ^*^p < 0.05, ^**^p < 0.01, and ^***^p < 0.001. Ctrl, Control; FGF23, fibroblast growth factor 23; TG, thiamet G (OGA inhibitor); OSMI-1, OGT inhibitor.

To determine the effect of FGF23 on the O-GlcNAc modification, we assessed the changes in global protein O-GlcNAc levels, and OGT and OGA protein expression in human bronchial epithelial cells (HBECs). FGF23 treatment of HBEC induced global changes in the O-GlcNAc modification of proteins (Figures 1A,B; Ctrl: 4.14 ± 0.25; FGF23: 5.23 ± 0.25, *p* = 0.0221), similar to the effects of the OGA inhibitor Thiamet G (Ctrl: 4.14 ± 0.25; TG: 7.22 ± 0.52, *p* = 0.0018), and opposite of the effects of OGT inhibition with OSMI-1 (FGF23: 5.23 ± 0.25; OSMI-1: 3.27 ± 0.43, *p* = 0.016). Consistent with the O-GlcNAc changes, both OGT and OGA levels were also increased following FGF23 stimulation (Figure 1A) with more of an increase in OGA than OGT [Figures 1C,D; (OGT: Ctrl: 0.35 ± 0.03; FGF23: 0.54 ± 0.032, *p* = 0.0059) and (OGA: Ctrl: 0.58 ± 0.06; FGF23: 1.5 ± 0.19, *p* = 0.003)], which has been shown in previous reports when O-GlcNAc levels are increased or treated with an OGA inhibitor (26).

### Modulation of O-GlcNAc is regulated through the PLCγ signaling pathway upon stimulation with FGF23

To determine whether FGF23 activates PLCγ (FGFR4-mediated), ERK (FGFR1-mediated), or both FGF23 signaling pathways in HBECs, we immunoblotted for total and phosphorylated PLCγ and ERK proteins Phosphorylation of PLCγ and total PLC was significantly increased without change in ERK phosphorylation following FGF23 administration [Figures 1E–G; (p-PLCγ: Ctrl: 0.38 ± 0.02; FGF23: 0.56 ± 0.02, *p* = 0.0054) and (ERK: Ctrl: 1.02 ± 0.02; FGF23: 0.96 ± 0.053, *p* = 0.375)]. Interestingly, OGA inhibition (TG) had a similar effect as FGF23 on the phosphorylation of PLCγ and ERK [Figures 1E–G; (p-PLCγ: Ctrl: 0.38 ± 0.02; TG: 0.51 ± 0.023, *p* = 0.022) and (ERK: Ctrl: 1.02 ± 0.02; TG: 0.90 ± 0.068, *p* = 0.21)], whereas OGT inhibition (OSM-I) did not have any affect on the phosphorylation of PLCγ or ERK levels [Figures 1E–G; (p-PLCγ: Ctrl: 0.38 ± 0.02; OSMI-1: 0.42 ± 0.01, *p* = 0.339) and (ERK: Ctrl: 1.02 ± 0.02; OSMI-1: 0.77 ± 0.12, *p* = 0.102)].

To determine whether the changes in O-GlcNAc following FGF23 are regulated through the PLCγ signaling pathway, we blocked PLCγ activation using a PLCγ inhibitor (U-73122), which has been shown to block FGF23 signaling through FGFR4 (27, 28). As shown in Figure 1H, O-GlcNAc was dose-dependently reduced following PLCγ inhibitor administration (Figure 1I: NT: 0.92 ± 0.090; 0.1 μM PLC inhibitor: 0.76 ± 0.07; 1.0 μM PLC inhibitor: 0.54 ± 0.02; *p* = 0.0095). In addition, OGT and OGA protein levels were decreased after PLCγ blockade (Figures 1H,J,K: OGT NT: 1.30 ± 0.15; 0.1 μM PLCγ inhibitor: 1.00 ± 0.08; 1.0 μM PLCγ inhibitor: 0.64 ± 0.10; *p* = 0.0073 and OGA NT: 1.08 ± 0.08; 0.1 μM PLCγ inhibitor: 0.91 ± 0.10; 1.0 μM PLCγ inhibitor: 0.57 ± 0.05; *p* = 0.0045) Altogether, these data suggest that FGF23 activates the PLCγ signaling pathway that regulates the O-GlcNAc changes observed in HBECs.

### Knockdown of OGT abrogates the O-GlcNAc modification of proteins and the effects of FGF23 signaling

**Figure 2:**
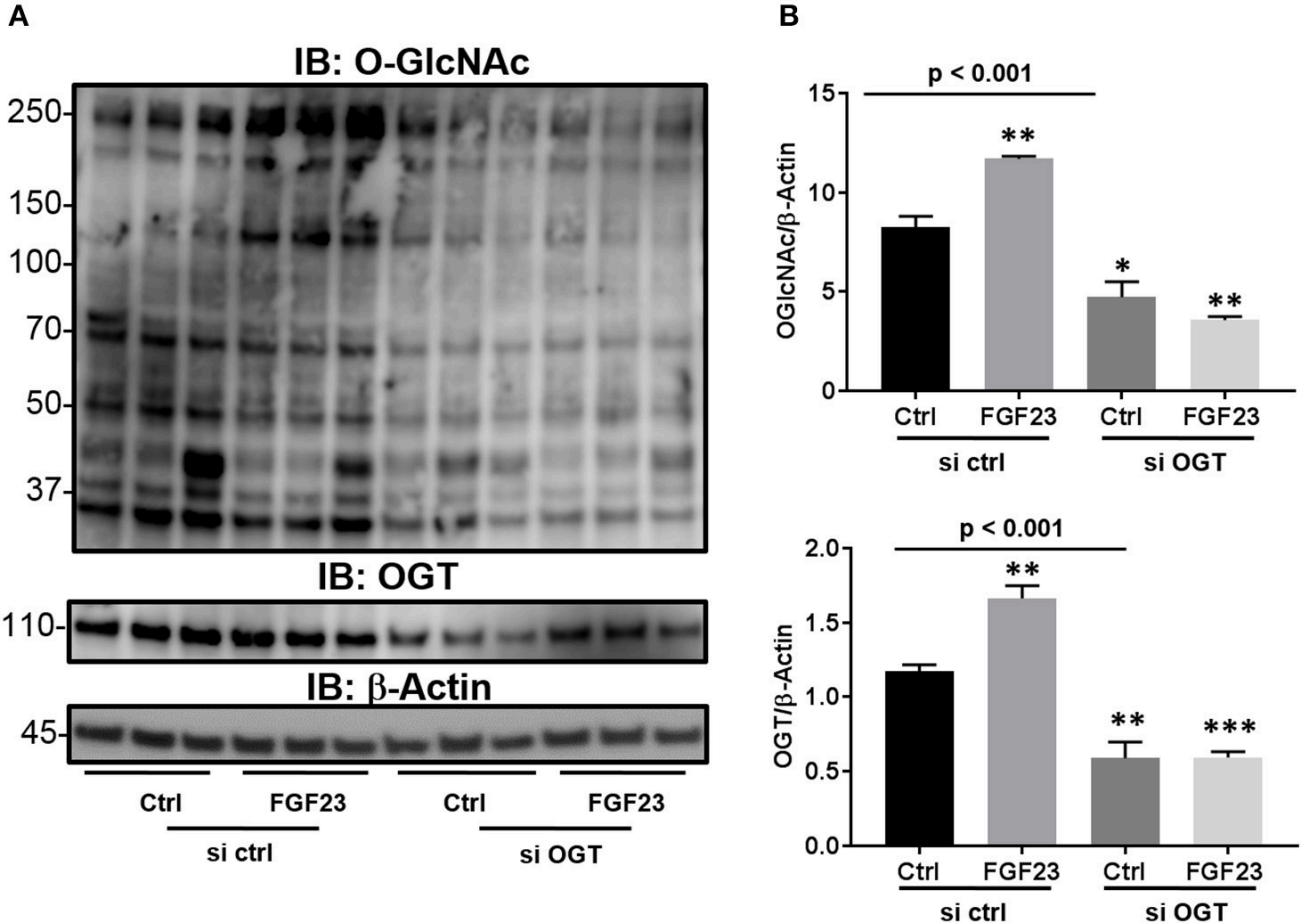
Knockdown of OGT reduces the O-GlcNAc modification of proteins. **(A)** Representative Immunoblots showing global O-GlcNAc, OGT, OGA, and β-Actin from HBECs treated as described or **(B)** HBECs were transfected with siRNA against OGT in the presence and absence of FGF23. Western blots were performed as triplicates of the same experiment. Ctrl, Control; FGF23, fibroblast growth factor 23; si ctrl, small interfering RNA control; and si OGT, small interfering RNA against OGT. Statistical analysis was done using ANOVA or Student's t-test showing means ± S.E.M. with ^*^p < 0.05, ^**^p < 0.01, and ^***^p < 0.001.

Previous reports have shown that there is an extracellular OGT (eOGT), which resides in the ER and transfers the GlcNAc moiety to epidermal growth factor-like domains (29). Interestingly, it has been shown to have high expression in the lung (30). Therefore, we wanted to determine whether the increase O-GlcNAc levels, stimulated by FGF23, is transferred by OGT and not the ER-resident eOGT in HBECs. To confirm that OGT is the sole enzyme responsible for O-GlcNAc transfer in these cells and affected by FGF23 signaling, we used siRNA targeted knockdown of OGT. As shown in Figure 2, FGF23 induced O-GlcNAc and OGT levels similar to Figures 1A–C [(O-GlcNAc: Ctrl: 8.23 ± 0.57; FGF23: 11.7 ± 0.13, *p* = 0.004) and (OGT: 1.18 ± 0.04; FGF23: 1.66 ± 0.09, *p* = 0.0068)]. Knockdown of OGT resulted in decreased O-GlcNAc levels in the presence or absence of FGF23 in HBECs [(O-GlcNAc: Ctrl: 8.23 ± 0.57; KD Ctrl: 4.73 ± 0.77; and KD FGF23: 3.57 ± 0.18, *p* < 0.001) and (OGT: Ctrl 1.18 ± 0.04; KD Ctrl: 0.59 ± 0.11; and KD FGF23: 0.60 ± 0.04, *p* < 0.001)]. Altogether, these results demonstrate that OGT is downstream of FGF23 signaling and solely responsible for the O-GlcNAc transfer. This data, along with Figure 1, suggests that FGF23 can modulate O-GlcNAc levels through a PLCγ-dependent signaling pathway, and can be inhibited by knockdown of OGT.

### Both FGF23 and OGA inhibition regulate IL-6 secretion in human bronchial epithelial cells

**Figure 3:**
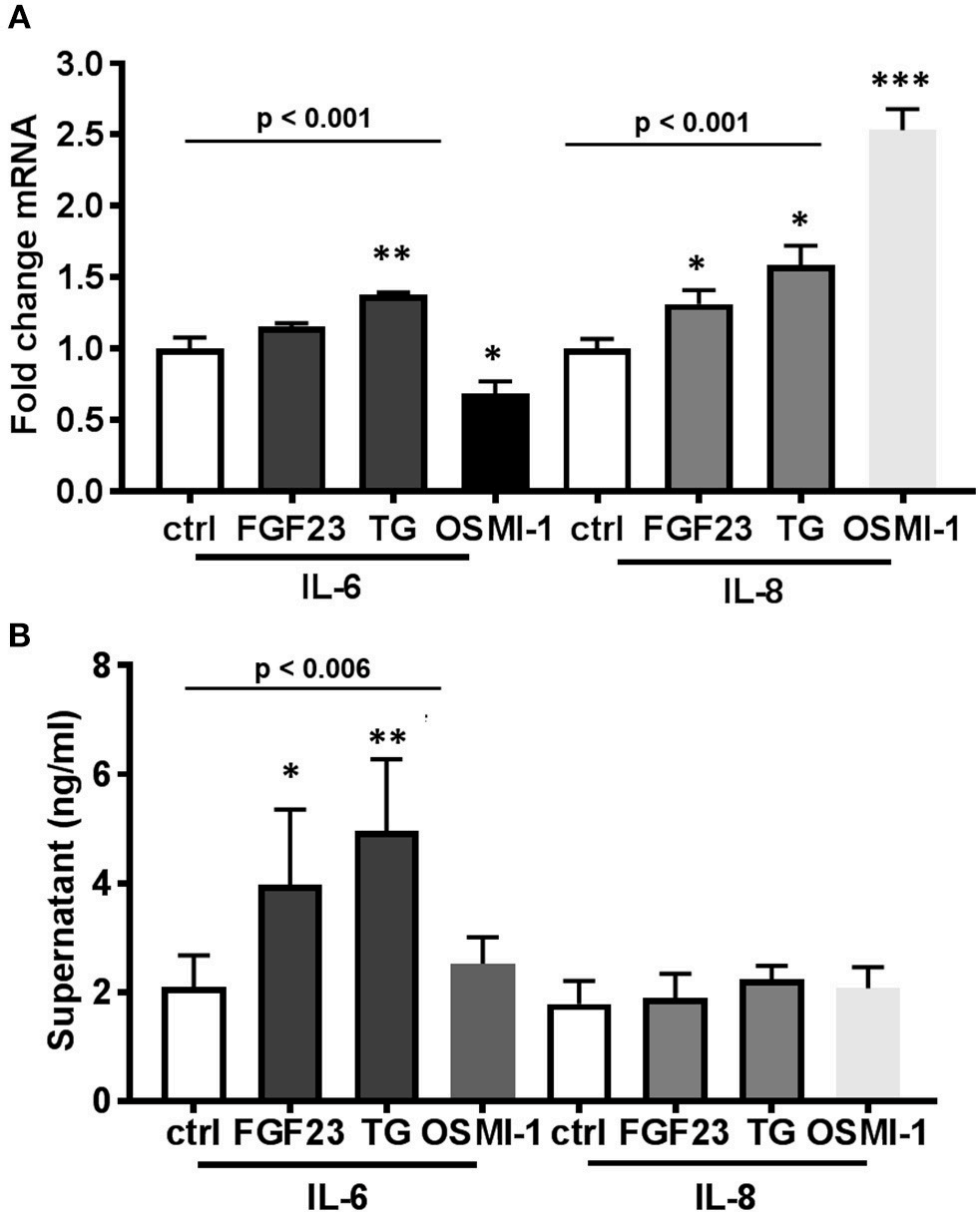
FGF23 and OGA inhibition both increase IL-6 secretion in human bronchial epithelial cells. **(A)** Bar graphs showing transcript analysis and fold change of IL-6 and IL-8 levels in HBECs treated in the presence or absence of FGF23, TG, or OSMI-1 for 24h. **(B)** IL-6 and IL-8 protein amounts determined by ELISA from conditioned media of HBECs. All experiments were done in triplicate and bar graphs shown respresent the mean ± S.E.M.(^*^p < 0.05; ^**^p < 0.01, and ^***^p < 0.001). Ctrl, Control; FGF23, fibroblast growth factor 23; TG, thiamet G (OGA inhibitor); OSMI-1, OGT inhibitor.

We previously demonstrated a significant positive correlation between circulating FGF23 and IL-6 levels in plasma of COPD patients (5). However, no studies have investigated the role of FGF23 and O-GlcNAc on inflammatory cytokine production. To determine effects of both FGF23 and O-GlcNAc levels on the inflammatory cytokine production in HBECs, we assessed mRNA and protein levels of IL-6 and IL-8. FGF23 stimulation led to a significant increase in IL-8 transcripts [Figure 3A; (IL-8: Ctrl: 1.0 ± 0.07; FGF23: 1.31 ± 0.10, *p* = 0.042)], whereas inhibition of OGA caused a significant increase in both IL-6 and IL-8 mRNA levels [Figure 3A; (IL-8: Ctrl: 1.0 ± 0.07; TG: 1.58 ± 0.14, *p* = 0.032) and (IL-6: Ctrl: 1.0 ± 0.08; TG: 1.38 ± 0.01, *p* = 0.013)]. Inhibition of O-GlcNAc transfer by OSMI-1 resulted in a reduction of IL-6 transcripts [Figure 3A; (IL-6: Ctrl: 1.0 ± 0.08; OSMI-1: 0.68 ± 0.09, *p* = 0.033)]; however, there was a significant increase in IL-8 mRNA expression upon OGT inhibition [Figure 3A; (IL-8: Ctrl: 1.0 ± 0.07; OSMI-1: 2.53 ± 0.15, *p* = 0.0006)]. As shown in Figure 3B, assessment of IL-6 protein secretion from conditioned media by ELISA showed a significant ~2-fold increase in IL-6 levels (IL-6: Ctrl: 2.10 ± 0.24; FGF23: 3.97 ± 0.62, *p* = 0.035) following FGF23 administration that was consistent with OGA inhibition [~2.5 fold, Figure 3B (IL-6: Ctrl: 2.10 ± 0.24; TG: 4.96 ± 0.93, *p* = 0.0035). Inhibition of OGT, though, did not show any effect compared to control. Surprisingly, IL-8 protein secretion, as assessed by ELISA, was not significantly different under any condition (Figure 3B, *p* = 0.435). These findings suggest that FGF23 and increased O-GlcNAc can lead to increased secretion of IL-6.

### FGF23 activates NFAT through O-GlcNAc augmentation in HBECs

**Figure 4:**
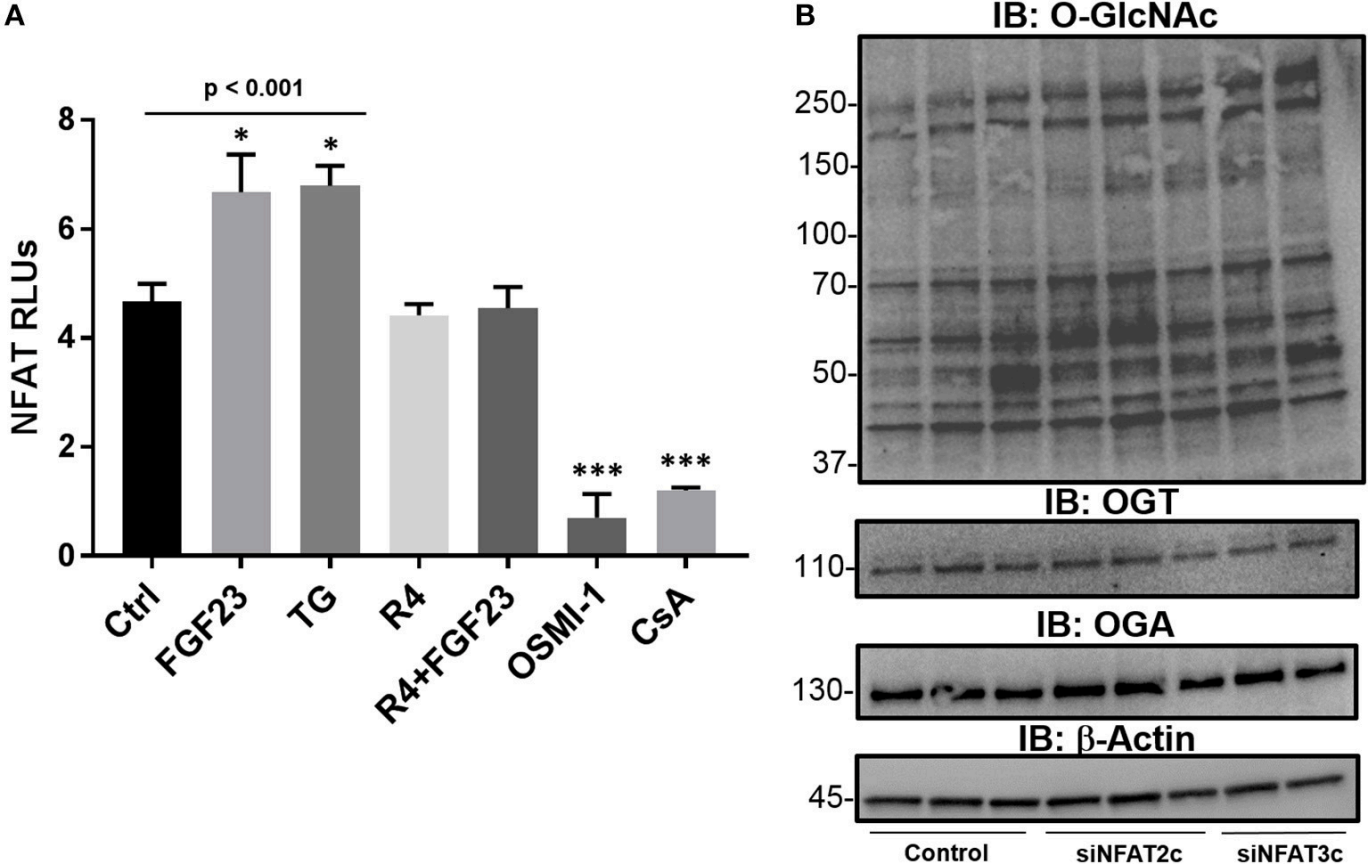
The NFAT activation is regulated by FGF23 and OGA inhibition (TG) and is downstream of O-GlcNAc regulation. **(A)** FGF23 and TG activate NFAT as assessed by a luciferase-based reporter gene assay in HBECs and is reduced to normal levels by an FGFR4 inhibitor (R4) or blocked by OSMI-1 similar to the effects of the NFAT activation inhibitor, cyclosporine (CsA). **(B)** Western blots of O-GlcNAc, OGT, and OGA following knockdown of NFAT2c and NFAT3c. Experiments were performed in triplicate. Statistical analysis was done using ANOVA or Student's *t*-test showing means ± S.E.M. with ^*^p < 0.05, and ^***^p < 0.001. Ctrl, Control; FGF23, fibroblast growth factor 23; TG, thiamet G (OGA inhibitor); R4, FGFR4 inhibitor; OSMI-1, OGT inhibitor; CsA, cyclosporine; and siNFAT, small interfering RNA against NFAT2c or 3c.

Previously published reports have shown an association between O-GlcNAc signaling and NFAT regulation in cardiomyocyte hypertrophy (13, 31) and in lymphocyte activation (32). In addition, we have recently shown that bronchial epithelial cells express NFAT2c and 3c as main isoforms and are activated by FGF23, which results in airway inflammation (5). To determine the role of FGF23 and O-GlcNAc in NFAT activation, we performed NFAT2c/3c activation assays using a luciferase-conjugated NFAT reporter gene in HBECs. As shown in Figure 4A, FGF23 significantly increased NFAT activation (Ctrl: 4.68 ± 0.32; FGF23: 6.68 ± 0.70, *p* = 0.035), *p* = 0.012), which is similar to our previous report (5). Inhibition of O-GlcNAc removal (TG) resulted in a similar increase in NFAT activation (Ctrl: 4.68 ± 0.32; TG: 6.80 ± 0.36, *p* = 0.01). Interestingly, blocking O-GlcNAc transfer significantly reduced NFAT activation (Ctrl: 4.68 ± 0.32; OSMI-1: 0.7 ± 0.16, *p* < 0.001), which was similar to cyclosporine (CsA; Ctrl: 4.68 ± 0.32; CsA: 1.2 ± 0.06, *p* = 0.003) and noticeably different compared to control, FGF23, and TG. Also in Figure 4A, FGFR4 blockade (R4) in the presence or absence of FGF23 inhibited NFAT activation consistent with the no treatment results [(R4: Ctrl: 4.68 ± 0.32; R4: 4.42 ± 0.21, *p* = 0.49 and FGF23+R4: Ctrl: 4.68 ± 0.32; FGF23+R4: 4.55 ± 0.39, *p* = 0.80)]. These data suggest that NFAT activation (through FGF23/FGFR4) is regulated by O-GlcNAc modulation in a similar fashion to FGF23 stimulation in HBECs.

To determine whether knockdown of NFAT effects O-GlcNAc modification of proteins, we silenced NFAT2c/3c using siRNA. As shown in Figure 4B, knockdown of NFAT2c/3c did not affect O-GlcNAc levels, or OGT/OGA protein expression. Altogether, these data combined suggests that NFAT activation is regulated through the FGF23 increase in O-GlcNAc levels, which lies upstream of NFAT in HBECs.

### Knockdown of NFAT2c or OGT leads to downregulation of IL-6 expression

**Figure 5:**
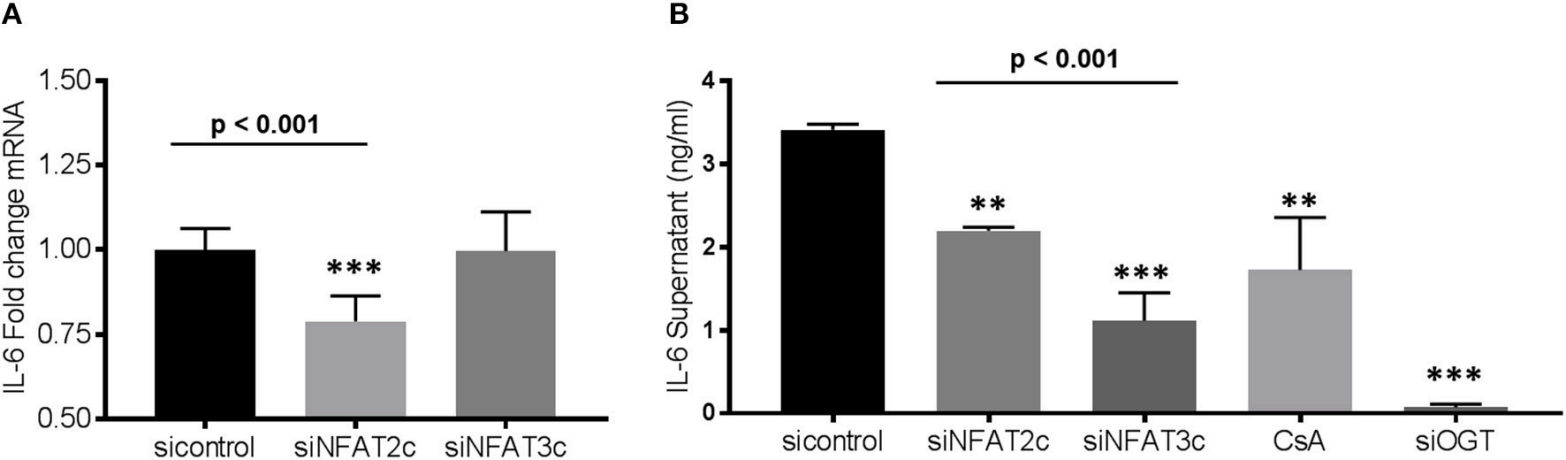
Gene silencing of NFAT2c leads to downregulation of IL-6 expression, while knockdown of both NFAT2c/3c results in reduced IL-6 secretion in HBECs. **(A)** Bar graphs showing transcript analysis and fold change of IL-6 levels following NFAT2/3C knockdown with siRNA in HBECs. **(B)** IL-6 protein level from conditioned media of HBECs as determined by ELISA. All experiments were done in triplicate and statistical analyses was done using ANOVA or Student's *t*-test showing means ± S.E.M. with ^**^p < 0.01 and ^***^p < 0.001. si control, small interfering RNA control; siNFAT, small interfering RNA against NFAT2c or 3c; small interfering RNA against OGT; and CsA, cyclosporin.

As shown above, administration of FGF23 or altering the O-GlcNAc levels in human bronchial epithelial cells resulted in changes in IL-6 expression (Figure 3). To determine whether knockdown of NFAT alters IL-6 expression, we silenced NFAT2c/3c and determined IL-6 transcript levels and protein secretion. As shown in Figure 5A, IL-6 mRNA was significantly lower in siNFAT2c compared to siNFAT3c and sicontrol (sicontrol: 1.0 ± 0.02; siNFAT2c: 0.79 ± 0.03; and siNFAT3c: 0.99 ± 0.04, *p* = 0.0008). At the protein level, IL-6 secretion was reduced following NFAT2c/3c knockdown and was consistent with CsA treatment (Figure 5B: sicontrol: 3.41 ± 0.06; siNFAT2c: 2.20 ± 0.04; siNFAT3c: 1.11 ± 0.33; and CsA: 1.72 ± 0.64, *p* = 0.0014). These results suggest that silencing NFAT2c/3c, which is downstream of FGF23 and O-GlcNAc, downregulates IL-6 in HBECs similar to blocking O-GlcNAc transfer (shown in Figure 2) or silencing OGT (Figure 5B: sicontrol: 3.41 ± 0.06; siOGT: 0.07 ± 0.04, *p* < 0.001).

## Discussion

In this report, we show that FGF23 can increase O-GlcNAc levels as well as OGT and OGA protein expression in HBECs. This effect seems to be a downstream target via the FGFR4/PLCγ signaling pathway (FGFR1/ERK signaling is not affected) since PLC blockade resulted in decreased O-GlcNAc following FGF23 adminstration (Figure 1H). Furthermore, both FGF23 and O-GlcNAc lead to increased secretion of IL-6, but not IL-8 (Figure 3). Conversely, reduction of O-GlcNAc levels (by OSMI-1) reduced IL-6 secretion. Upon assessing effects of the NFAT activation in HBECs (Figure 4), we found that FGF23 activated NFAT, which is consistent with our previous report (5). In line with this, O-GlcNAc modulation resulted in either an increase in NFAT activation (by blocking O-GlcNAc removal) or a decrease in NFAT upon inhibiting O-GlcNAc transfer (OSMI-1) or FGF23 signaling (through blockade of FGFR4). Interestingly, knockdown of NFAT 2c or 3c did not affect the O-GlcNAc levels or OGA/OGT expression (Figure 4B). However, NFAT2c silencing did affect both IL-6 expression and secretion, while NFAT3c knockdown only affected IL-6 secretion (Figure 5).

**Figure 6:**
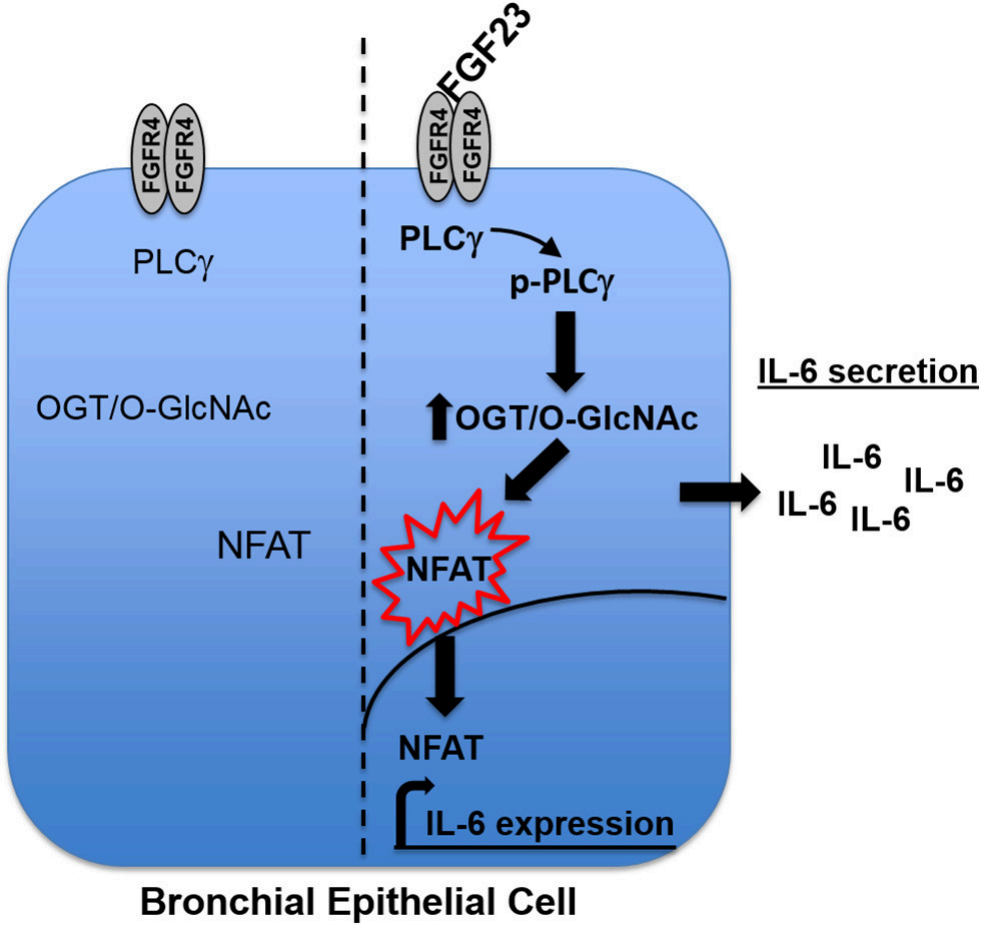
Model. Upon binding to FGFR4 in human bronchial epithelial cells, FGF23 stimulates the phosphorylation of PLCγ, which increases the O-GlcNAc modification of proteins. The increase in O-GlcNAc results in NFAT activation and translocation from the cytoplasm to the nucleus where it drives the expression of IL-6 and subsequent secretion out of the cell.

In our results, there were discrepancies in the mRNA expression of IL-6 compared to IL-8 with the different treatments (Figures 3, 5). In addition, there was higher IL-6 protein secretion compared to IL-8, which did not correlate with the respective mRNA levels. This was also similar with the IL-6 results for NFAT3c in Figure 5. These discrepancies in the mRNA expression and protein levels of cytokines have been documented in other studies (33). Therefore, caution should be used when interpreting mRNA expression as a proxy to protein levels. In addition, we cannot rule out the fact that the increased levels of IL-6 (or no change in levels of IL-8) in our study may be due to altered cytokine uptake/turnover following treatments. Nevertheless, these data combined suggest that FGF23 stimulation of O-GlcNAc levels is upstream of NFAT signaling in HBECs and regulates the secretion of the pro-inflammatory cytokine IL-6 (Figure 6).

FGF23 has been characterized as a hormonal regulator of circulating phosphate and vitamin D levels as well as a prognostic risk factor for cardiovascular mortality in patients with chronic kidney disease (28, 34–36). The role of FGF23 role as an inflammatory facilitator has also been recently studied (5, 37, 38). Interestingly, it may be involved in several metabolic processes, including glucose and fat metabolism. For example, FGF23 was shown to contribute to insulin sensitivity in obese adolescents (39) and was altered in vitamin D deficient patients following an oral glucose load (40). Based on these findings, administration of FGF23 may alter metabolic pathways that are involved in glucose dysregulation and/or inflammation. The O-GlcNAc modification has long been studied and defined as cellular nutrient/stress sensor and the connection between the FGF23 and O-GlcNAc levels is plausible based on these previous reports and our results.

The O-GlcNAc modification regulates nuclear and cytosolic protein function and cellular signaling. Previous reports have shown a pro- and anti-inflammatory role for O-GlcNAc (23, 41), and this biphasic effect is dependent on different cell types and diseases. Previous reports have also shown that glucosamine activation of the HBP attenuates NFκB activation in chondrocytes (42) or IL-1β mediation chondrocyte activation (43). In addition, inhibition of the NFκB pathway by O-GlcNAc has been shown in acute vascular injury models (44). On the other hand, the O-GlcNAc modification has been shown to activate NFκb under increase glucose concentrations in vascular smooth muscle cells in diabetes and obesity, suggesting a pro-inflammatory phenotype (45). This pro-inflammatory phenotype has been shown in other reports where HBP flux augments the oxidative stress pathways and the expression of other pro-inflammatory markers vascular cell adhesion molecule-1 (VCAM-1), IL6, IL-1β, TNFα, and NFκB (46). To our knowledge, we are the first to show the link between FGF23 and O-GlcNAc regulating IL-6 expression (Figure 3).

Several reports have shown that “outside-in” signaling can be modulated by O-GlcNAc. In T cell activation, antigen peptide binding to the T cell receptor have been shown to increase the O-GlcNAc modification of proteins that were associated with inflammatory cytokine production and cellular proliferation (47). In bone morphogenic protein (BMP) signaling on osteoblast differentiation, hyperglycemic conditions or activators of the HBP were shown to alter the O-GlcNAc levels and affect osteogenic differentiation (48) suggesting a role for glucose dysregulation on normal BMP function and signaling. In another report, FGF signaling was also shown to be altered by loss of function of a gene that encodes an enzyme in the HBP, which resulted in defective O-GlcNAc modification of the FGFR (49, 50). This was shown to impair FGF mediated migration of mesodermal and tracheal cells during fly development. In addition, Miura, T. and colleagues demonstrated that O-GlcNAc modification of PKCζ blocks the signaling effects of FGF4, which resulted in the maintenance of an ESC undifferentiated state (51). A role for the O-GlcNAc modification was also shown for PLC inactivation and subsequent reduction of IP3 and Ca2+ mobilization in myoblast even in response to bradykinin (52). Similar to these findings, we show that FGF23 signaling, through PLCγ, increases the O-GlcNAc modification of proteins that may be involved in inflammatory cytokine production in HBECs (Figures 1–3). Interestingly, we also observed an increase in total PLCγ protein expression (Figure 1). The increase in total PLCγ expression has been shown in other inflammatory associated complications, including cancer and skin conditions (53) that may be associated with altered FGF23 levels (54–56) and is consistent with our findings. Collectively, we show that FGF23 signaling, through PLCγ, increases the O-GlcNAc modification of proteins that may be involved in inflammatory cytokine production in HBECs (Figures 1–3).

As stated above, NFAT signaling has also been linked to inflammatory cytokine production in hepatocytes, angiogenesis, cardiomyocyte hypertrophy, and many other biological processes (27, 28, 37, 57). In addition, T- and B-lymphocytes activation, which has been documented to be regulated by NFAT (32), can be regulated by the O-GlcNAc modification, which may be required for its nuclear translocation. A similar phenomenon was shown in cardiomyocyte hypertrophy where the activation of NFAT has been linked to increased O-GlcNAc modification (13, 58). Our previous report suggested a role for FGF23 activation of NFAT in the airway (5). However, no experiments have been done to determine the role of O-GlcNAc on NFAT activation through FGF23. Based on our results, combined with our previously published data, we put forth a model (Figure 6), whereby FGF23 regulates NFAT activation through the modulation of O-GlcNAc (Figures 4, 5), and stimulates IL-6 expression and secretion in HBECs (Figures 1–3).

Our findings in this report are the first to describe a role for FGF23 in the augmentation of O-GlcNAc levels. In addition, the role for FGF23 in the activation of NFAT through O-GlcNAc stimulation increases our knowledge of the molecules that may be involved in the process. The impact of O-GlcNAc by way of FGF23 will open new avenues for research in lung diseases associated with chronic airway inflammation such as COPD, cystic fibrosis, and asthma as well as metabolic disorders including diabetes and heart failure.

## Author contributions

SK and JB conceived, designed, and analyzed all experiments and wrote the manuscript. EH, SH, SB, JG, RD, RZ, and HW performed experiments that were conceptualized and guided by JB and SK.

### Conflict of interest statement

The authors declare that the research was conducted in the absence of any commercial or financial relationships that could be construed as a potential conflict of interest.

## References

[1] Richter B Faul C. FGF23 actions on target tissues-with and without klotho. Front Endocrinol. (2018) 9:189. 10.3389/fendo.2018.00189 29770125 PMC5940753

[2] Itoh N Ohta H Konishi M. Endocrine FGFs: evolution, physiology, pathophysiology, and pharmacotherapy. Front Endocrinol. (2015) 6:154. 10.3389/fendo.2015.00154 26483756 PMC4586497

[3] Gutierrez OM Mannstadt M Isakova T Rauh-Hain JA Tamez H Shah A. Fibroblast growth factor 23 and mortality among patients undergoing hemodialysis. N Engl J Med. (2008) 359:584–92. 10.1056/NEJMoa0706130 18687639 PMC2890264

[4] Shimada T Urakawa I Isakova T Yamazaki Y Epstein M Wesseling-Perry K. Circulating fibroblast growth factor 23 in patients with end-stage renal disease treated by peritoneal dialysis is intact and biologically active. J Clin Endocrinol Metab. (2010) 95:578–85. 10.1210/jc.2009-1603 19965919 PMC2840849

[5] Krick S Grabner A Baumlin N Yanucil C Helton S Grosche A. Fibroblast growth factor 23 and Klotho contribute to airway inflammation. Eur Respir J. (2018) 52:1800236. 10.1183/13993003.00236-2018 29748308 PMC6044452

[6] Krick S Baumlin N Aller SP Aguiar C Grabner A Sailland J. Klotho inhibits interleukin-8 secretion from cystic fibrosis airway epithelia. Sci Rep. (2017) 7:14388. 10.1038/s41598-017-14811-0 29085059 PMC5662572

[7] Yang TT Suk HY Yang X Olabisi O Yu RY Durand J. Role of transcription factor NFAT in glucose and insulin homeostasis. Mol Cell Biol. (2006) 26:7372–87. 10.1128/MCB.00580-06 16908540 PMC1636854

[8] Liu Q Chen Y Auger-Messier M Molkentin JD. Interaction between NFkappaB and NFAT coordinates cardiac hypertrophy and pathological remodeling. Circ Res. (2012) 110:1077–86. 10.1161/CIRCRESAHA.111.260729 22403241 PMC3341669

[9] Siamakpour-Reihani S Caster J Bandhu Nepal D Courtwright A Hilliard E Usary J. The role of calcineurin/NFAT in SFRP2 induced angiogenesis–a rationale for breast cancer treatment with the calcineurin inhibitor tacrolimus. PLoS ONE (2011) 6:e20412. 10.1371/journal.pone.0020412 21673995 PMC3108822

[10] Noren DP Chou WH Lee SH Qutub AA Warmflash A Wagner DS. Endothelial cells decode VEGF-mediated Ca2+ signaling patterns to produce distinct functional responses. Sci Signal. (2016) 9:ra20. 10.1126/scisignal.aad3188 26905425 PMC5301990

[11] Buse MG. Hexosamines, insulin resistance, and the complications of diabetes: current status. Am J Physiol Endocrinol Metab. (2006) 290:E1–8. 10.1152/ajpendo.00329.2005 16339923 PMC1343508

[12] Sage AT Walter LA Shi Y Khan MI Kaneto H Capretta A. Hexosamine biosynthesis pathway flux promotes endoplasmic reticulum stress, lipid accumulation, and inflammatory gene expression in hepatic cells. Am J Physiol Endocrinol Metab. (2010) 298:E499–511. 10.1152/ajpendo.00507.2009 19952345

[13] Facundo HT Brainard RE Watson LJ Ngoh GA Hamid T Prabhu SD. O-GlcNAc signaling is essential for NFAT-mediated transcriptional reprogramming during cardiomyocyte hypertrophy. Am J Physiol Heart Circ Physiol. (2012) 302:H2122–30. 10.1152/ajpheart.00775.2011 22408028 PMC3362113

[14] Love DC Hanover JA. The hexosamine signaling pathway: deciphering the “O-GlcNAc code”. Sci STKE (2005) 2005:re13. 10.1126/stke.3122005re13 16317114

[15] Marshall S Bacote V Traxinger RR. Discovery of a metabolic pathway mediating glucose-induced desensitization of the glucose transport system. Role of hexosamine biosynthesis in the induction of insulin resistance. J Biol Chem. (1991) 266:4706–12. 2002019

[16] Wells L Vosseller K Hart GW. A role for N-acetylglucosamine as a nutrient sensor and mediator of insulin resistance. Cell Mol Life Sci. (2003) 60:222–8. 10.1007/s000180300017 12678487 PMC11138838

[17] Varki A Cummings RD Esko JD Freeze HH Stanley P Bertozzi CR Essentials In Glycobiology. Cold Spring Harbor, NY: Cold Spring Harbor Laboratory Press (2009). 20301239

[18] Myslicki JP Belke DD Shearer J. Role of O-GlcNAcylation in nutritional sensing, insulin resistance and in mediating the benefits of exercise. Appl Physiol Nutri Metab. (2014) 39:1205–13. 10.1139/apnm-2014-0122 25203141

[19] Sodi VL Bacigalupa ZA Ferrer CM Lee JV Gocal WA. Nutrient sensor O-GlcNAc transferase controls cancer lipid metabolism via SREBP-1 regulation. Oncogene (2017) 37:924–34. 10.1038/onc.2017.395 29059153 PMC5814337

[20] Slawson C Housley MP Hart GW. O-GlcNAc cycling: how a single sugar post-translational modification is changing the way we think about signaling networks. J Cell Biochem. (2006) 97:71–83. 10.1002/jcb.20676 16237703

[21] Hart GW. Three decades of research on O-GlcNAcylation - a major nutrient sensor that regulates signaling, transcription and cellular metabolism. Front Endocrinol. (2014) 5:183. 10.3389/fendo.2014.00183 25386167 PMC4209869

[22] Vaidyanathan K Durning S Wells L. Functional O-GlcNAc modifications: implications in molecular regulation and pathophysiology. Crit Rev Biochem Mol Biol. (2014) 49:140–63. 10.3109/10409238.2014.884535 24524620 PMC4912837

[23] Banerjee PS Lagerlof O Hart GW. Roles of O-GlcNAc in chronic diseases of aging. Mol Aspects Med. (2016) 51:1–15. 10.1016/j.mam.2016.05.005 27259471

[24] Barnes JW Tian L Heresi GA Farver CF Asosingh K Comhair SA. O-linked beta-N-acetylglucosamine transferase directs cell proliferation in idiopathic pulmonary arterial hypertension. Circulation (2015) 131:1260–8. 10.1161/CIRCULATIONAHA.114.013878 25663381 PMC4390469

[25] Schneider CA Rasband WS Eliceiri KW. NIH Image to ImageJ: 25 years of image analysis. Nat Methods (2012) 9:671–5. 10.1038/nmeth.2089 22930834 PMC5554542

[26] Zhang Z Tan EP VandenHull NJ Peterson KR Slawson C. O-GlcNAcase expression is sensitive to changes in O-GlcNAc homeostasis. Front Endocrinol. (2014) 5:206. 10.3389/fendo.2014.00206 25520704 PMC4249489

[27] Grabner A Amaral AP Schramm K Singh S Sloan A Yanucil C. Activation of cardiac fibroblast growth factor receptor 4 causes left ventricular hypertrophy. Cell Metab. (2015) 22:1020–32. 10.1016/j.cmet.2015.09.002 26437603 PMC4670583

[28] Faul C Amaral AP Oskouei B Hu MC Sloan A Isakova T. FGF23 induces left ventricular hypertrophy. J Clin Invest. (2011) 121:4393–408. 10.1172/JCI46122 21985788 PMC3204831

[29] Sakaidani Y Nomura T Matsuura A Ito M Suzuki E Murakami K. O-Linked-N-acetylglucosamine on extracellular protein domains mediates epithelial cell–matrix interactions. Nat Commun. (2011) 2:583. 10.1038/ncomms1591 22158438

[30] Sakaidani Y Ichiyanagi N Saito C Nomura T Ito M Nishio Y. O-linked-N-acetylglucosamine modification of mammalian Notch receptors by an atypical O-GlcNAc transferase Eogt1. Biochem Biophys Res Commun. (2012) 419:14–19. 10.1016/j.bbrc.2012.01.098 22310717

[31] McLarty JL Marsh SA Chatham JC. Post-translational protein modification by O-linked N-acetyl-glucosamine: its role in mediating the adverse effects of diabetes on the heart. Life Sci. (2013) 92:621–7. 10.1016/j.lfs.2012.08.006 22985933 PMC3528804

[32] Golks A Tran TT Goetschy JF Guerini D Requirement for O-linked N-acetylglucosaminyltransferase in lymphocytes activation. EMBO J. (2007) 26:4368–79. 10.1038/sj.emboj.7601845 17882263 PMC2034663

[33] Shebl FM Pinto LA García-Piñeres A Lempicki R Williams M Harro C. Comparison of mRNA and protein measures of cytokines following vaccination with human papillomavirus-16 L1 virus-like particles. Cancer Epidemiol Biomark Prev. (2010) 19:978–81. 10.1158/1055-9965.EPI-10-0064 20332253 PMC2852493

[34] Quarles LD. Role of FGF23 in vitamin D and phosphate metabolism: implications in chronic kidney disease. Exp Cell Res. (2012) 318:1040–8. 10.1016/j.yexcr.2012.02.027 22421513 PMC3336874

[35] Wolf M. Update on fibroblast growth factor 23 in chronic kidney disease. Kidney Int. (2012) 82:737–47. 10.1038/ki.2012.176 22622492 PMC3434320

[36] Gutierrez OM Wolf M Taylor EN. Fibroblast growth factor 23, cardiovascular disease risk factors, and phosphorus intake in the health professionals follow-up study. Clin J Am Soc Nephrol. (2011) 6:2871–8. 10.2215/CJN.02740311 22034506 PMC3255372

[37] Singh S Grabner A Yanucil C Schramm K Czaya B Krick S. Fibroblast growth factor 23 directly targets hepatocytes to promote inflammation in chronic kidney disease. Kidney Int. (2016) 90:985–96. 10.1016/j.kint.2016.05.019 27457912 PMC5065745

[38] Grabner A Mazzaferro S Cianciolo G Krick S Capelli I Rotondi S. Fibroblast growth factor 23: mineral metabolism and beyond. Contrib Nephrol. (2017) 190:83–95. 10.1159/000468952 28535521 PMC5997251

[39] Wojcik M Dolezal-Oltarzewska K Janus D Drozdz D Sztefko K Starzyk JB. FGF23 contributes to insulin sensitivity in obese adolescents - preliminary results. Clin Endocrinol. (2012) 77:537–40. 10.1111/j.1365-2265.2011.04299.x 22103239

[40] Ursem SR Vervloet MG Buttler RM Ackermans MT Oosterwerff MM Eekhoff MV. The interrelation between FGF23 and glucose metabolism in humans. J Diabetes Complications (2018) 32:845–50. 10.1016/j.jdiacomp.2018.06.013 29996975

[41] Hirata Y Nakagawa T Moriwaki K Koubayashi E Kakimoto K Takeuchi T. Augmented O-GlcNAcylation alleviates inflammation-mediated colon carcinogenesis via suppression of acute inflammation. J Clin Biochem Nutr. (2018) 62:221–9. 10.3164/jcbn.17-106 29892160 PMC5990405

[42] Imagawa K de Andres MC Hashimoto K Pitt D Itoi E Goldring MB. The epigenetic effect of glucosamine and a nuclear factor-kappa B (NF-kB) inhibitor on primary human chondrocytes–implications for osteoarthritis. Biochem Biophys Res Commun. (2011) 405:362–7. 10.1016/j.bbrc.2011.01.007 21219853 PMC3937866

[43] Shikhman AR Kuhn K Alaaeddine N Lotz M. N-acetylglucosamine prevents IL-1 beta-mediated activation of human chondrocytes. J Immunol. (2001) 166:5155–60. 10.4049/jimmunol.166.8.5155 11290798

[44] Yao D Xu L Xu O Li R Chen M Shen H. O-Linked beta-N-acetylglucosamine modification of A20 enhances the inhibition of NF-kappaB (Nuclear Factor-kappaB) activation and elicits vascular protection after acute endoluminal arterial injury. Arterioscler Thromb Vasc Biol. (2018) 38:1309–20. 10.1161/ATVBAHA.117.310468 29622561

[45] Yang WH Park SY Nam HW Kim DH Kang JG Kang ES. NFkappaB activation is associated with its O-GlcNAcylation state under hyperglycemic conditions. Proc Natl. Acad Sci USA. (2008) 105:17345–50. 10.1073/pnas.0806198105 18988733 PMC2582288

[46] James LR Tang D Ingram A Ly H Thai K Cai L. Flux through the hexosamine pathway is a determinant of nuclear factor kappaB- dependent promoter activation. Diabetes (2002) 51:1146–56. 10.2337/diabetes.51.4.1146 11916938

[47] Lund PJ Elias JE Davis MM. Global analysis of O-GlcNAc glycoproteins in activated human T cells. J Immunol. (2016) 197:3086–98. 10.4049/jimmunol.1502031 27655845 PMC5055199

[48] Sun C Shang J Yao Y Yin X Liu M Liu H. O-GlcNAcylation: a bridge between glucose and cell differentiation. J Cell Mol Med. (2016) 20:769–81. 10.1111/jcmm.12807 26929182 PMC4831356

[49] Ghabrial AS. A sweet spot in the FGFR signal transduction pathway. Sci Signal. (2012) 5:pe1. 10.1126/scisignal.2002789 22253260 PMC3362922

[50] Mariappa D Sauert K Marino K Turnock D Webster R van Aalten DM. Protein O-GlcNAcylation is required for fibroblast growth factor signaling in Drosophila. Sci Signal. (2011) 4:ra89. 10.1126/scisignal.2002335 22375049 PMC3660836

[51] Miura T Kume M Kawamura T Yamamoto K Hamakubo T Nishihara S. O-GlcNAc on PKCzeta Inhibits the FGF4-PKCzeta-MEK-ERK1/2 pathway via inhibition of PKCzeta phosphorylation in mouse embryonic stem cells. Stem Cell Rep. (2018) 10:272–86. 10.1016/j.stemcr.2017.11.007 29249667 PMC5768893

[52] Kim YH Song M Oh YS Heo K Choi JW Park JM. Inhibition of phospholipase C-beta1-mediated signaling by O-GlcNAc modification. J Cell Physiol. (2006) 207:689–96. 10.1002/jcp.20609 16538662

[53] Kim MJ Kim E Ryu SH Suh PG. The mechanism of phospholipase C-gamma1 regulation. Exp Mol Med. (2000) 32:101–9. 10.1038/emm.2000.18 11048639

[54] Saito T Fukumoto S. Fibroblast growth factor 23 (FGF23) and disorders of phosphate metabolism. Int J Pediatr Endocrinol. (2009) 2009:496514. 10.1186/1687-9856-2009-496514 19956747 PMC2775677

[55] Aukes K Forsman C Brady NJ Astleford K Blixt N Sachdev D. Breast cancer cell-derived fibroblast growth factors enhance osteoclast activity and contribute to the formation of metastatic lesions. PLoS ONE (2017) 12:e0185736. 10.1371/journal.pone.0185736 28968431 PMC5624603

[56] Kim HJ Kim KH Lee J Oh JJ Cheong HS Wong EL. Single nucleotide polymorphisms in fibroblast growth factor 23 gene, FGF23, are associated with prostate cancer risk. BJU Int. (2014) 114:303–10. 10.1111/bju.12396 24053368

[57] Suehiro J Kanki Y Makihara C Schadler K Miura M Manabe Y. Genome-wide approaches reveal functional vascular endothelial growth factor (VEGF)-inducible nuclear factor of activated T cells (NFAT) c1 binding to angiogenesis-related genes in the endothelium. J Biol Chem. (2014) 289:29044–59. 10.1074/jbc.M114.555235 25157100 PMC4200259

[58] Gelinas R Mailleux F Dontaine J Bultot L Demeulder B Ginion A. AMPK activation counteracts cardiac hypertrophy by reducing O-GlcNAcylation. Nat Commun. (2018) 9:374. 10.1038/s41467-017-02795-4 29371602 PMC5785516

